# A case of tape infection 19 years after insertion of a tension‐free vaginal tape sling

**DOI:** 10.1002/iju5.12440

**Published:** 2022-03-28

**Authors:** Tomofumi Watanabe, Tomoko Sako, Yusuke Tominaga, Takuya Sadahira, Takanori Sekito, Atsushi Takamoto, Kohei Edamura, Yasuyuki Kobayashi, Koichiro Wada, Motoo Araki

**Affiliations:** ^1^ Department of Urology Okayama University Graduate School of Medicine, Dentistry and Pharmaceutical Sciences Okayama Japan; ^2^ Department of Urology Fukuyama City Hospital Fukuyama Hiroshima Japan; ^3^ Department of Urology Shimane University Faculty of Medicine Matsue Shimane Japan

**Keywords:** asymptomatic dysuria, stress urinary incontinence, tension‐free vaginal tape, urinary tract infection

## Abstract

**Introduction:**

Tape infection after insertion of tension‐free vaginal tape is a well‐known but rare complication. We report a patient who experienced a subcutaneous abscess 19 years after the surgery.

**Case presentation:**

A 41‐year‐old woman presented with fever and lower abdominal pain. She had undergone tension‐free vaginal tape insertion for stress urinary incontinence 19 years prior. She had asymptomatic dysuria. After an abscess incision and 1‐week treatment with antibiotics, she underwent surgery to remove the tape and the abscess without complications.

**Conclusion:**

Tension‐free Vaginal Tape insertion could be a potential risk of asymptomatic dysuria, resulting in urinary tract infection. In this case, removal of tape was necessary for controlling subcutaneous abscess resulting from the presence of tension‐free vaginal tape.

**Abbreviations & Acronyms**
CT: computed tomography
SUI: stress urinary incontinence
TVT: tension‐free vaginal tape

**Keynote message**

We report a patient who experienced a subcutaneous abscess 19 years after the surgery, indicating the importance of appropriate follow‐up after Tension‐free Vaginal Tape to detect long‐term complications such as dysuria or mesh erosion. We recommend tape removal and antibiotic therapy when Tension‐free Vaginal Tape infection is suspected.

## Introduction

The treatment of SUI includes physical therapy, medication, and surgery.^1^ Insertion of TVT, one of the minimally invasive techniques used for women with SUI, is widely performed because of its efficacy, but its complications cannot be ignored. The main complications of TVT surgery are bladder stones, bladder perforation (3.5%), tape erosion (0.2%), urinary tract infections (0.7–17%), pelvic hematomas (3.4%), and in rare cases, intestinal, and vascular injury.^2^, ^3^, ^4^ Infection is a potential complication of TVT insertion, occurring at a rate of 0.7%.^4^ It may occur in the perioperative period, although there are reports of infection as long‐term complications of TVT, with a urethral fistula or tape erosions. It is extremely rare for a subcutaneous abscess to form without a urethral fistula present. We present a patient with tape infection 19 years after TVT surgery; she had a subcutaneous abscess and a paravesical abscess but no evidence of tape erosion or urethral fistula. She was successfully cured with surgical tape removal.

## Case presentation

**Fig. 1:**
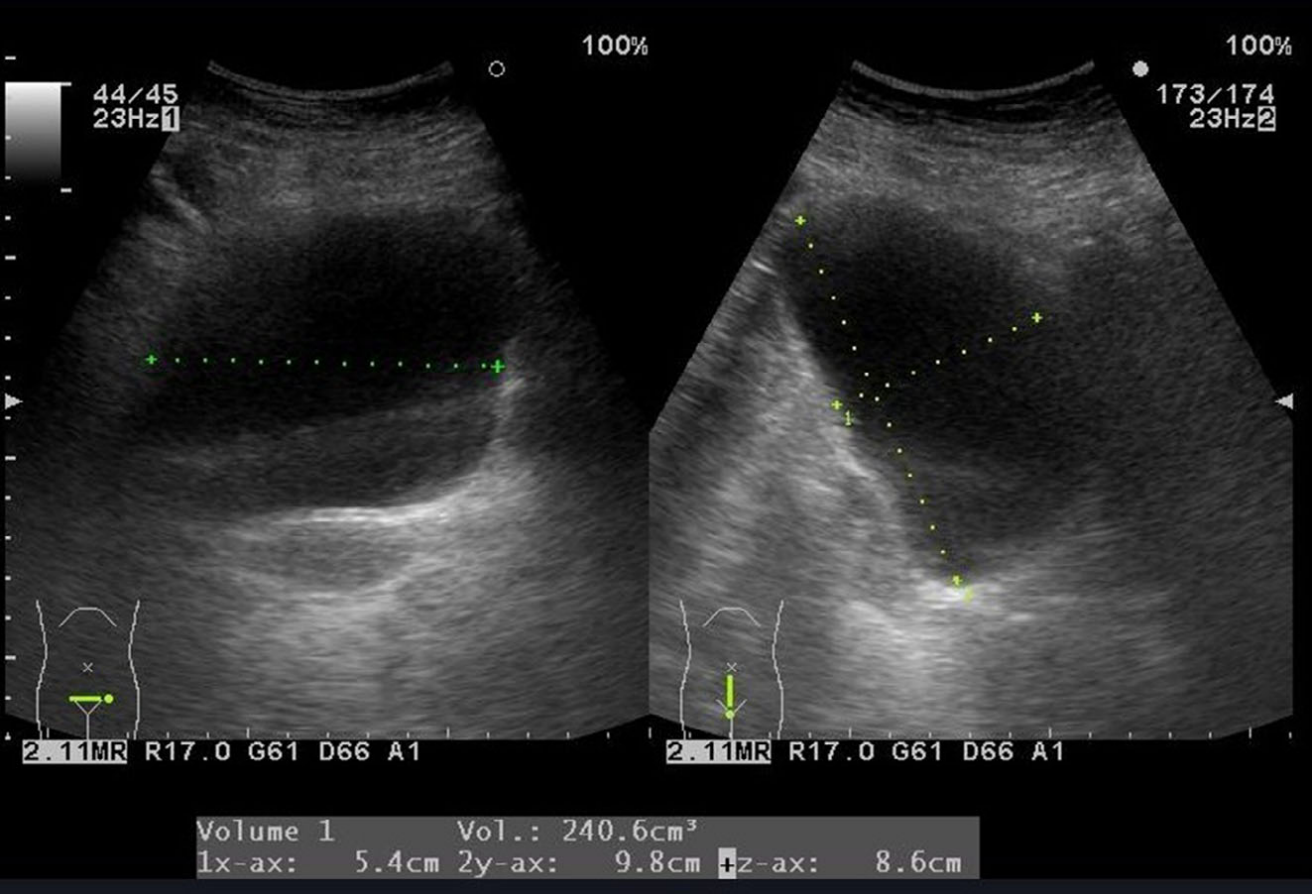
Abdominal ultrasonography showing a hypoechoic area in the right suprapubic area and a postvoid residual urine volume of 240 mL. [Colour figure can be viewed at wileyonlinelibrary.com]

A 41‐year‐old woman presented to our department in January 2021 with urinary frequency and right suprapubic pain. Two weeks before her first visit, she was diagnosed with cystitis and outpatient antibiotic therapy was performed with levofloxacin(500 mg/day) for several days by her family doctor. She had undergone TVT insertion for severe SUI 19 years prior under proper counseling and consent, but her compliance was so poor that she quit coming soon after the initial TVT insertion. Her body mass index was 28.3. Her medical history was significant for schizophrenia and type 2 diabetes mellitus. There was no history of other surgery or pelvic radiation. Her medication included glimepiride, sitagliptin phosphate hydrate, pioglitazone hydrochloride, metformin hydrochloride, urapidil, biperiden hydrochloride, paroxetine hydrochloride hydrate, risperidone, etizolam, brotizolam, and carbamazepine. Physical examination revealed induration and tenderness of the right suprapubic area but no fever. Abdominal ultrasonography showed a hypoechoic area in the same region; her postvoid residual urine volume was 240 mL (Fig. 1), indicating the presence of asymptomatic dysuria. Urinalysis revealed pyuria (urinary white blood cell count: 30–49/HPF). Her urine culture was negative. Cystoscopy, transvaginal ultrasonography, and vaginal examination revealed no evidence of a urethrovaginal fistula or tape erosion into the bladder or vagina.

**Fig. 2:**
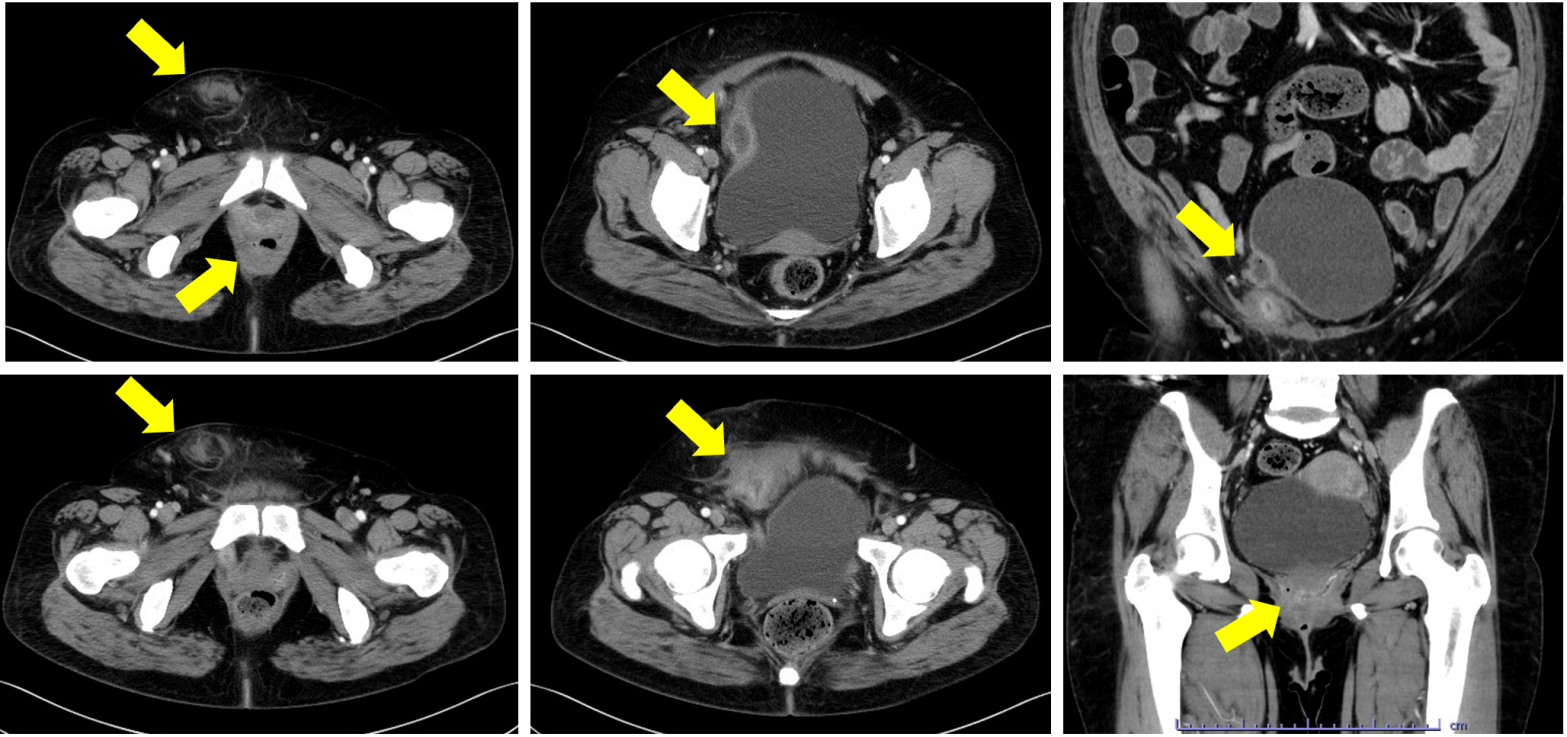
Contrast‐enhanced CT showing low‐density areas with ring enhancement suggestive of the abscess (arrow) in the right inguinal region, abdominal wall, lateral bladder cavity, and perivaginal area along the TVT insertion site. [Colour figure can be viewed at wileyonlinelibrary.com]

We initiated outpatient antibiotic therapy with levofloxacin (500 mg/day), but the patient returned the following day with a fever and worsening pain. The right suprapubic area was red, hot, and indurated. Laboratory examination showed increased levels of C‐reactive protein (14.15 mg/dL) and high white blood cell count (14.2 × 10^9^/L); hemoglobin A1c and blood glucose levels were normal. Contrast‐enhanced CT showed low‐density areas with ring enhancement, suggestive of abscess, in the right inguinal region, abdominal wall, lateral bladder cavity, and perivaginal area; these areas corresponded to the route of the TVT implant (Fig. 2). She was admitted to the hospital for incision and drainage, and her antibiotic was changed to meropenem (2.0 mg/day, 6 days). Urine and blood cultures from admission were negative. When the abscess culture revealed *Streptococcus anginosus*, we de‐escalated antibiotic therapy to ampicillin/sulbactam (6.0 g/day, 12 days).

**Fig. 3:**
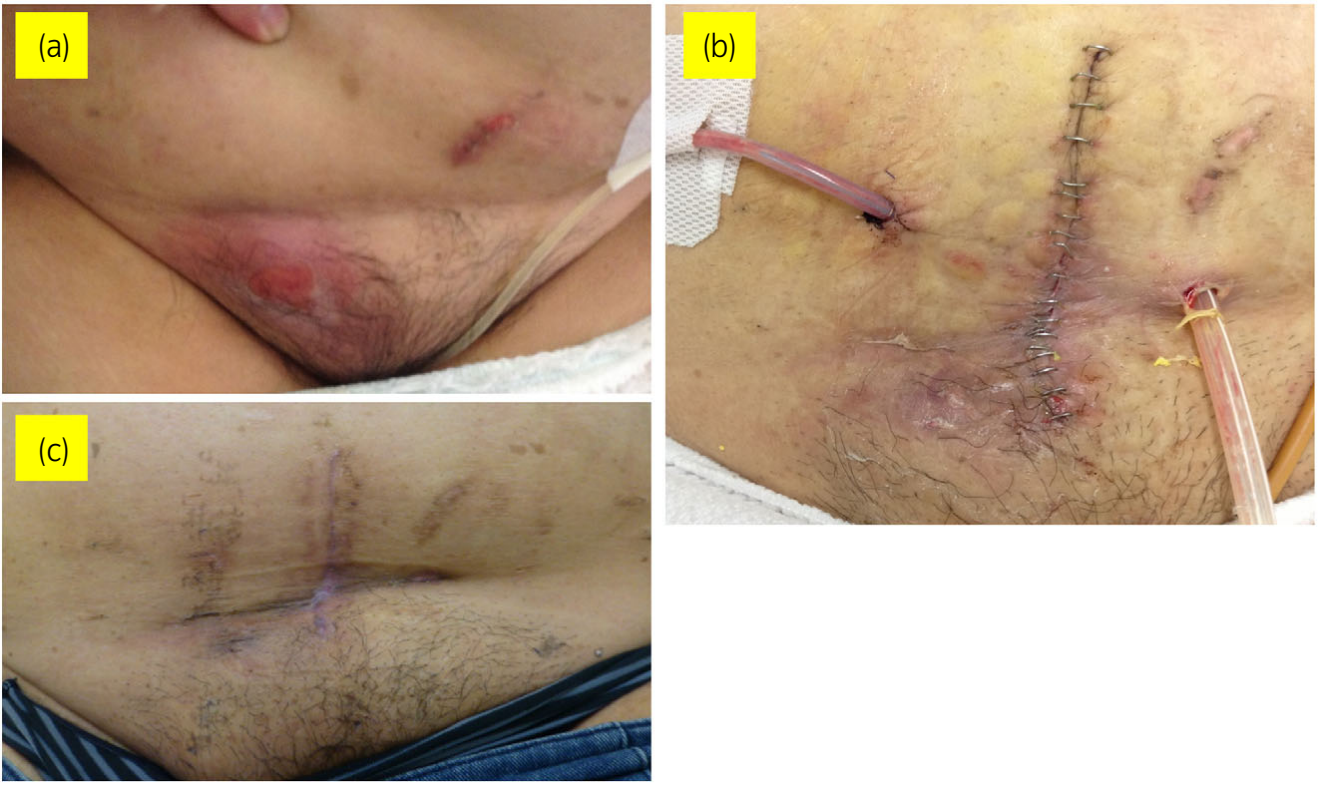
Pictures of a healing wound. (a) One day after incision and drainage; (b) 1 day after surgery, the pelvic and subcutaneous drainage tubes are present; (c) 1 month after surgery. [Colour figure can be viewed at wileyonlinelibrary.com]

**Fig. 4:**
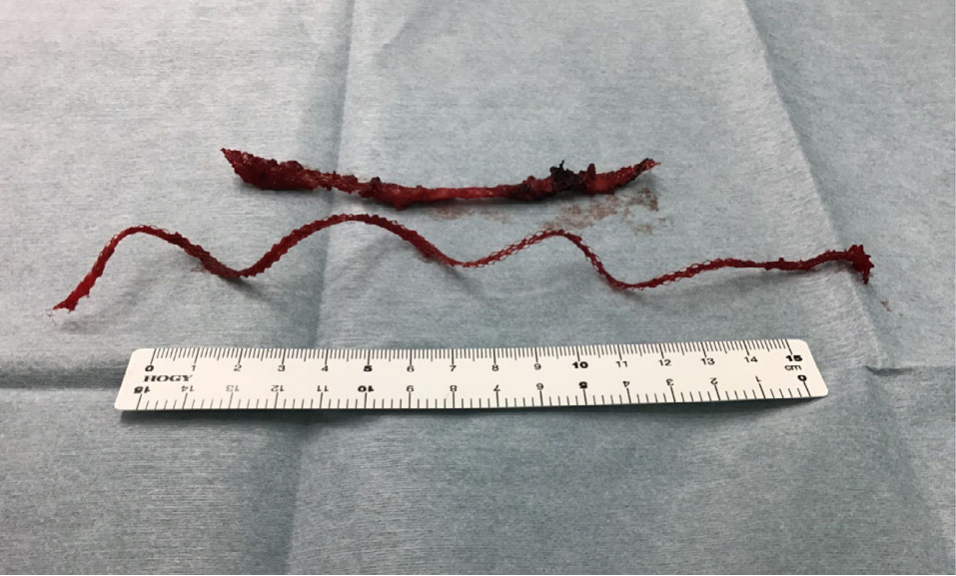
Removed TVT implant. [Colour figure can be viewed at wileyonlinelibrary.com]

Although her fever and tenderness improved, the redness and swelling did not completely resolve (Fig. 3a). On the 10th day after admission, we performed surgical abscess drainage and removed the TVT implant. We reached the suburethral portion of the tape through a vaginal approach, cut the tape in the middle, and detached it from the urethra with blunt dissection technique. We also made a midline vertical incision to expand the retropubic space. We successfully located both ends of the tape; the abscess spread all around the tape, and both sides of the tape were too close to the bladder wall even though the CT showed the abscess more severely on the right side. We excised granulation tissue, drained the abscesses noted on CT, and removed the implant from the vagina (Fig. 4). Intraoperative cystoscopy showed a defect in the muscular layer of the left bladder sidewall and muscular layer repair was performed. At last, we placed pelvic and subcutaneous drainage tubes (Fig. 3b). However, there was no evidence of tape erosion or urethral fistula.

The drainage tubes were removed on the fifth and sixth postoperative days, and the patient was discharged from the hospital 1 week after surgery, after switching to amoxicillin hydrate (750 mg/day, 6 days). The postvoid residual volume on the day of discharge was normal. The patient's postoperative course has been uneventful, without recurrence of infection. The surgical incisions are well‐healed (Fig. 3c), and her incontinence has not returned.

## Discussion

Long‐term complications of TVT surgery include the formation of a urethrovaginal fistula, tape erosion, and tape infection.^5^, ^6^, ^7^ Few patients with fistula or abscess formation after TVT surgery have been cured by conservative treatment alone, suggesting that surgical treatment is necessary. Early detection of mesh erosion or inappropriate mesh position is difficult, but Tunn reported a patient who had early detection of TVT erosion by introital ultrasound; this emphasizes the role of introital ultrasound in the diagnostic evaluation of functional disturbances occurring after TVT surgery.^8^

Risk factors for fistula formation or mesh erosion after TVT surgery include pelvic radiation, malignancy, and menopause.^5^ Although our patient did not have any of these factors, her compliance was poor, and she did have several risk factors for neurogenic bladder: diabetes, a mental disorder, and antipsychotic medication use. Her abscess contained *Streptococcus anginosus*, which could cause complicated urinary tract infections.^9^ Thus, we suspect that chronic dysuria, diabetes, and inappropriate mesh position might have caused chronic urinary tract infection, which subsequently spread along the course of the tape rather than infection from the skin or vagina. Chene reports a 31% incidence of symptomatic dysuria 5 years after TVT surgery and a 52% incidence of asymptomatic dysuria.^10^ These figures suggest that asymptomatic dysuria is more common than symptomatic dysuria, illustrating the importance of follow‐up care to assess dysuria.

In conclusion, TVT surgery should be avoided as much as possible for young people since it could be a risk factor of abscess formation, and appropriate follow‐up after TVT is necessary to evaluate postoperative functional disturbances and to deal quickly with dysuria, inappropriate mesh position, and mesh erosion. We recommend tape removal and antibiotic therapy when TVT infection is suspected.

## Author Contributions

Tomofumi Watanabe: Conceptualization; investigation; visualization; writing – original draft. Tomoko Sako: Conceptualization; data curation; writing – review and editing. Yusuke Tominaga: Investigation; writing – review and editing. Takuya Sadahira: Conceptualization; supervision; writing – review and editing. Takanori Sekito: Writing – review and editing. Atsushi Takamoto: Investigation; writing – review and editing. Kohei Edamura: Writing – review and editing. Yasuyuki Kobayashi: Investigation; writing – review and editing. Koichiro Wada: Data curation; investigation; writing – review and editing. Motoo Araki: Supervision.

## Conflict of interest

The authors declare no conflict of interest.

## Approval of the research protocol by an Institutional Reviewer Board

Not applicable.

## Informed consent

All informed consent was obtained from the subject.

## Registry and the Registration No. of the study/trial

Not applicable.
